# Dietary *Platycodon grandiflorus* Attenuates Hepatic Insulin Resistance and Oxidative Stress in High-Fat-Diet Induced Non-Alcoholic Fatty Liver Disease

**DOI:** 10.3390/nu12020480

**Published:** 2020-02-14

**Authors:** Weixin Ke, Pan Wang, Xuehua Wang, Xiaolu Zhou, Xiaosong Hu, Fang Chen

**Affiliations:** 1 College of Food Science and Nutritional Engineering, National Engineering Research Center for Fruit and Vegetable Processing, Key Laboratory of Fruit and Vegetable Processing, Ministry of Agriculture, Engineering Research Centre for Fruit and Vegetable Processing, Ministry of Education, China Agricultural University, Beijing 100083, China; weixin.ke@unil.ch (W.K.);; 2 Department of Fundamental Microbiology, University of Lausanne, 1015 Lausanne, Switzerland; 3 Department of Bioengineering and Therapeutic Sciences, University of California, San Francisco, CA 94158, USA

**Keywords:** *Platycodon grandiflorus*, NAFLD, high-fat diet, insulin resistance, oxidative stress

## Abstract

The root of *Platycodon grandiflorus* (PG), with hepatoprotective and anti-oxidation effects, has a long history of being used as food and herbal medicine in Asia. However, the mechanism of PG against non-alcoholic fatty liver disease (NAFLD) is still not clear. The aim of this study was to investigate the mechanism of PG suppressing the development of NAFLD induced by a high-fat diet (HFD) in mice. Male C57BL/6J mice were fed with either a standard chow diet or a HFD, either supplemented with or without PG, for 16 weeks. Serum lipids, liver steatosis, oxidative stress and insulin sensitivity were determined. Expressions or activities of hepatic enzymes in the related pathways were analyzed to investigate the mechanisms. PG significantly reduced HFD-induced hepatic injury and hyperlipidemia, as well as hepatic steatosis via regulating phosphorylation of acetyl-CoA carboxylase (p-ACC) and expression of fatty acid synthase (FAS). In addition, PG ameliorated oxidative stress by restoring glutathione (GSH) content and antioxidant activities, and improved insulin sensitivity by regulating the PI3K/Akt/GSK3β signaling pathway. Our data showed that dietary PG have profound effects on hepatic insulin sensitivity and oxidative stress, two key factors in the pathogenesis of NAFLD, demonstrating the potential of PG as a therapeutic strategy for NAFLD.

## 1. Introduction

Non-alcoholic fatty liver disease (NAFLD) is the most common chronic liver disease, characterized by fat accumulation in the absence of significant alcohol consumption and has become a serious health problem with a prevalence among 24% of the global adult population [1,2]. Modern unhealthy diets, characterized by chronic excessive energy intake, like a high-fat diet (HFD), are one of the major causes of NAFLD, which can increase the accumulation of fat and aggravate the burden on antioxidation, insulin sensitivity and immune system in liver.

The pathogenesis of NAFLD is complex and is currently explained best by the two-hit-hypothesis [3]. The first hit involves insulin resistance and lipid accumulation in hepatocytes, which leads to hepatic steatosis and increases the vulnerability of liver. The second hit is constituted by many factors induced by first hit, including oxidative stress, subsequent lipid peroxidation, and inflammation.

Insulin resistance as one of the major risk factors in the pathogenesis of NAFLD triggers oxidative stress, inflammation, and promotes exacerbation of NAFLD, further leading to non-alcoholic steatohepatitis (NASH) [3]. Insulin regulates blood glucose levels mainly through the PI3K/Akt/ GSK3β signaling pathway [4]. Insulin can bind to and activate the insulin receptor, which promotes phosphorylation of insulin receptor substrates, leading to the activation of PI3K and downstream enzymes to regulate glucose transport and metabolism [5,6]. Oxidative stress, caused by reactive oxygen species (ROS) accumulation, could be induced by hepatic steatosis and insulin resistance [7]. Moreover, previous studies have shown that excessive oxidative stress could in turn exacerbate the development of insulin resistance [8,9,10]. Thus, agents that could attenuate insulin resistance and oxidative stress might be effective in treating NAFLD.

Because of the absence of an established pharmacotherapy, current recommendations for the treatment of NAFLD emphasize the management of lifestyle, including physical exercise and dietary modifications [11]. Functional fruits and vegetables with natural bioactive ingredients often possess strong anti-obesity, antioxidant and anti-inflammatory abilities, which may be ideal treatments for NAFLD and related metabolic disorders [12,13].

The root of *Platycodon grandiflorus (JACQ.) A. DC.* (Campanulaceae) (PG), commonly known as *Jiegeng* in China, *Doraji* in Korea, and *Kikyo* in Japan, has long been used as a typical functional food and a traditional herbal medicine. In mice, PG exhibits various pharmacological activities against obesity [14,15], inflammation [16,17], hypolipidemia and oxidative stress [18,19] because of some bioactive components, like saponins and polysaccharides. Previous studies have shown that saponins from PG have beneficial effects on alcoholic hepatitis and NASH via mediating lipogenesis [20,21], lipid peroxidation and anti-inflammation [22] in mice. However, the effects of PG on NAFLD, especially the insulin resistance and oxidative stress of NAFLD remain unclear.

Therefore, we designed this study to explore the effects and mechanisms of PG on hepatic insulin resistance and oxidative stress in HFD-induced NAFLD mice. Moreover, recent studies have emphasized on the crucial roles of insoluble or indigestible components of plants in their beneficial functions [23,24]. Thus, we use the whole root of PG in this study rather than extractions or specific components focused on by most previous studies.

## 2. Materials and Methods

### 2.1. Preparation of PG

Naturally dried PG roots were purchased from Chifeng, Inner Mongolia Autonomous Region, China. A total of 1 kg PG were cut into pieces and freeze-dried for 48 h, followed by milling by a commercial hand-carry milling machine to make PG powder, which were subsequently kept at 4 °C in a moisture-controlled cabinet. On the day of use, the PG powder was mixed with sterile physiological saline at a concentration of 200 mg/mL. All the other chemicals used in the study were at least of analytical grade.

### 2.2. Animal Experiment

Six-week old C57BL/6J mice purchased from Beijing Vital River Laboratory Animal Technology Co., Ltd. (Beijing, China) were facility with 12 h day and night cycles at 22 °C in a standard specific-pathogen-free (SPF) condition with food and water. Mice were fed with normal chow diet for acclimatization for 1 week and were randomly divided into four groups (8 per group) for a 16-week experiment: standard chow diet (SD, containing 10% fat by energy) or high fat diet group (HFD, containing 60% fat by energy), and each of them was also separated into two groups, either administered with PG by intragastric gavage (SDPG and HFPG) or with sterile physiological saline as control. The ingredients and energy densities of the experimental diets are shown in Supplementary Table S1. PG was intragastric administrated at a dose of 2 g/kg body weight daily. *Chinese Pharmacopoeia 2015* recommended the dose of PG intake for adults is 3–10 g/day, which can be translated to 0.615–2.05 g/kg body weight for mice model by body surface area normalization method [25]. Previous studies on mice model using PG saponins (composed of around 2% in PG) usually at a dose of 10–200 mg/kg body weight, which equals to a 0.5–10 g/kg of whole PG root. Thus, we decided to intragastric administrate PG at a dose of 2 g/kg body weight. At the end of the experiment, all mice were starved for 12 h before being sacrificed. Blood samples were collected and immediately centrifuged at 3000 rpm for 20 min to obtain the serum. Livers were promptly dissected out, weighted, immersed in liquid nitrogen and stored at −80 °C for further analysis or freshly immersed in formalin for histological analysis. All animal experiments were approved by the Animal Care Committee of China Agricultural University and according to the Guide for the Care and Use of Laboratory Animals (National Institutes of Health (NIH), Bethesda, MD, USA).

### 2.3. Glucose Tolerance Test

At week 15, glucose tolerance tests (ipGTTs) were conducted. Mice were starved over-night before injected by glucose (1.0 g/kg, intraperitoneally). Blood samples were collected from the tip of the tail vein at 0, 15, 30, 60, 90, and 120 min to measure blood glucose using a glucometer (Accu-chek, Roche, Switzerland). Areas under the curve (AUC) above baseline were calculated as index of glucose tolerance.

For the measurements of serum insulin levels, mice were starved for 16 h and fasted blood glucose levels were detected. A mouse insulin enzyme-linked immunosorbent assay kit (ELISA) (Alpco, USA) was used for determining the fasting insulin concentration. Homeostatic model assessment of insulin resistance (HOMA-IR) was calculated by the formula below:

HOMA-IR = [fasting glucose (mM) × fasting insulin (mU/L)]/22.5

### 2.4. Biochemical Analysis

Serum total triacylglycerol (TG), total cholesterol (TC), low-density lipoprotein cholesterol (LDL-C) alanine transaminase (ALT), high-density lipoprotein cholesterol (HDL-C) and aspartate transaminase (AST) were determined by commercial kits (Applygen Technologies Inc., Beijing, China) and measured by an OLYMPUS Automatic Biochemical Analyzer (AU480, Japan Olympus Corporation, Tokyo, Japan). Hepatic TG and TC were measured by commercial kits (BeyoTime, Nantong, China).

### 2.5. Measurement of Antioxidant Defenses

The total hepatic GSH contents were determined by a commercial assay kit (Cayman Chemical, Ann Arbor, MI, USA). GSH in samples were reacted with 5,5-dithiobis-(2-nitrobenzoic acid) before measuring by a fluorescence spectrophotometer at 405 nm.

Hepatic superoxide dismutase (SOD) activity evaluation was based on the inhibition of superoxide anion-mediated reduction from nitro blue tetrazolium (NBT) to NBT-diformazan, measured by a fluorescence spectrophotometry at 550 nm. One unit (U) of SOD activity was determined as the amount of enzyme required to suppress half of NBT reduction.

Hepatic catalase (CAT) and glutathione peroxidase (GSH-Px) activities were measured by commercial kits (Nanjing Jiancheng Bioengineering Institute, Nanjing, China). CAT activity was determined by the capability of H_2_O_2_ consumption and detected by a spectrophotometer at 240 nm. GSH-Px activity was measured by the capability of oxidizing glutathione (GSH) into glutathione disulfide (GSSG).

### 2.6. Histological Analysis

Liver tissues fixed in formalin were embedded in paraffin and sections (5–7 µm) were stained with hematoxylin and eosin (H&E) or Oil-Red O. Microscopy analysis was performed by a Zeiss Observer (Carl Zeiss, Oberkochen, Germany) at 200× magnification, and images were taken by an Olympus digital camera (Nikon DS-L1, Tokyo, Japan). Steatosis were scored based on the percentage of the total area affected into the following categories: 0 (<5%), 1 (5–30%), 2 (31–50%), 3 (50–75%) and 4 (>75%).

### 2.7. Real-Time Quantitative PCR

Target gene expressions were assessed using RT-qPCR on liver mRNA, which was isolated by a Trizol reagent (Invitrogen, Waltham, MA, USA), purified by a RNeasy Mini Kit (Qiagen, Venlo, Netherlands) and reverse transcribed with a FastQuant RT Kit (TianGen, Beijing, China). Template cDNA (1.5 μL) was mixed with 0.5 μL primers, 5 μL SYBR Green I Master Mix (Roche Diagnostics, Basel, Switzerland) and 3 μL water. PCRs were performed in triplicates on a LightCycler 480 Real-Time PCR system following this program: initial denaturation at 95 °C for 10 min, 35 PCR cycles of 95 °C for 10 s, 60 °C for 30 s, 72 °C for 15 s, followed by a melting curve. The used primers are listed in Supplementary Table S2. Relative quantification was calculated by the comparative 2–^ΔΔCT^ method with 5 biological replicates and was normalized against β-actin gene expression. Mean expression level of SD mice was set at a value of 1 for data normalization.

### 2.8. Western Blot

Liver tissues were homogenized with a RIPA buffer (Beyotime, Shanghai, China) supplemented with 1% protease inhibitor and 1% phosphatase inhibitor (Sigma-Aldrich, St. Louis, MO, USA) followed by centrifugation for 15 min at 12 000 rpm, 4 °C and the supernatant was used to quantification by a bicinchoninic acid protein assay kit (Thermo scientific, Waltham, MA, USA). The proteins were separated by 10% SDS-PAGE gels, then transferred onto polyvinylidene fluoride (PVDF) membranes (Millipore, Bedford, MA, USA) and blotted by Tris based 5% fat-free milk with 0.1% Tween 20 for 2 h under room temperature. The blots were processed for immunodetection with primary antibodies overnight at 4 °C followed by washing for three times and incubation with a horseradish peroxidase conjugated secondary antibody for 1 h under room temperature. Finally, enhanced chemiluminescence reagent (Millipore, Bedford, MA, USA) was added onto the membranes and the immunoblotted bands densitometry (optical density) were quantified by ImageJ software (National Institutes of Health, Bethesda, MD, USA). Mean expression level of SD mice was set at a value of 1 for data normalization.

The primary antibody against β-actin (No. ab8227) was purchased from Abcam (Cambridge, UK). The primary antibodies against acetyl-CoA carboxylase (ACC) (No. 3676), phospho-ACC (p-ACC) (Ser79) (No. 11818), fatty acid synthase (FAS) (No.3180), NAD(P)H: quinone acceptor oxidoreductase 1 (NQO1) (No. 62262), heme oxygenase 1(HO-1) (No.70081), phosphoinositide 3-kinases (PI3K) (No. 4257), phospho-PI3K (p-PI3K) (Tyr458) (No. 17366), protein kinase B (Akt) (No. 4691), phosphorylated-Akt (p-Akt) (Ser473) (No. 4060), glycogen synthase kinase 3β (GSK3β) (No. 12456), phospho-GSK3β (Ser9) (No. 5558), glycogen synthase (GS) (No. 3886), phospho-GS (Ser641) (No. 3891) and horseradish peroxidase-conjugated anti-rabbit secondary antibody (No. 7074) were purchased from Cell Signaling Technology (Danvers, MA, USA).

### 2.9. Statistical Analysis

All data were presented as means ± SEM. Comparisons among groups were performed by one-way ANOVA followed by Tukey’s post hoc test (SPSS v20.0, Chicago, IL, USA) and graphed by GraphPad Prism (San Diego, CA, USA). *p* < 0.05 was considered as statistically significant.

## 3. Results

### 3.1. PG Attenuated Lipid Accumulation and Liver Injuries in HFD-Fed Mice

**Figure 1:**
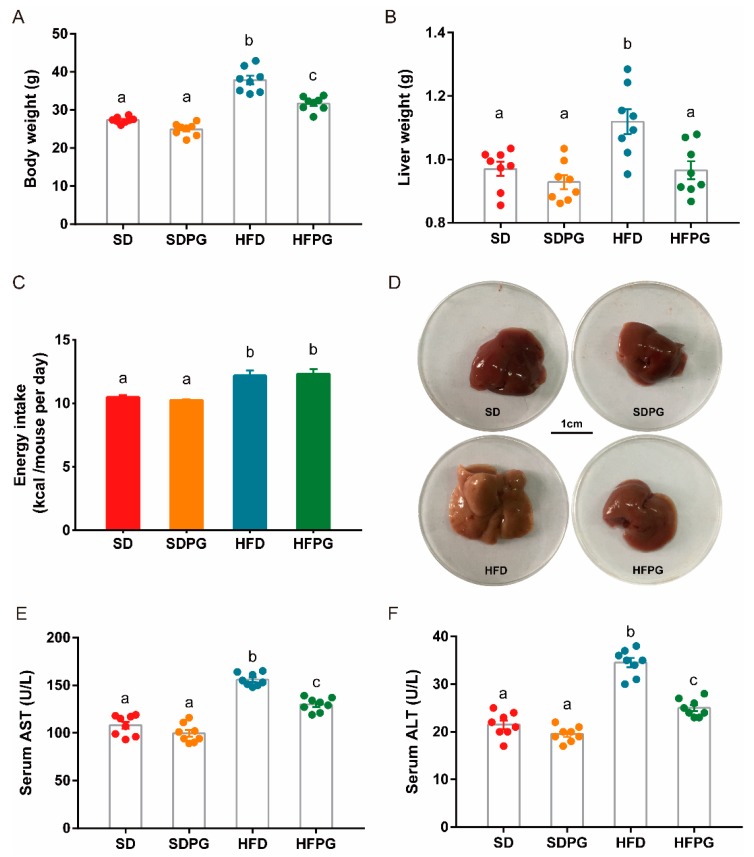
PG reduced fat accumulation and liver injury in HFD mice: (**A**) body weight and (**B**) liver weight at the end of the experiment. (**C**) Mean energy intake per mouse per day. (**D**) Typical liver morphological images (scale: 1 cm). (**E**) Serum AST and (**F**) ALT levels. a, b, c means in the same bar without a common letter differ at *p* < 0.05.

We showed that compared to the SD mice, HFD had increased body weight and liver weight (Figure 1A,B), although there was no significantly difference in body weight across the four groups at the beginning of the experiment (data not shown). PG supplementation significantly reduced body and liver weight of mice fed HFD compared with SD (Figure 1A,B). The average daily energy intake of mice within same diet group was no significant difference, although as expected, mice fed on HFD got more energy intake than mice fed on SD (Figure 1C).

Likewise, we found PG significantly reduced the HFD-induced hyperlipidemia (Table 1). Compared to HFD mice, HFPG mice showed marked decrease in plasma TC, TG, HDL-C, LDL-C levels, and lower ratios of LDL-C to HDL-C levels, which were at similar levels in both SD and SDPG mice. Furthermore, the mice fed with HFD for 16 weeks had discolored livers compared with SD mice, which were ameliorated by PG (Figure 1D). Moreover, serum AST and ALT levels were considered as liver damage markers, which were decreased by PG in HFPG mice, further demonstrating the protective effect of PG against HFD-induced hepatic injury (Figure 1E,F).

### 3.2. PG Reduced Steatosis in Liver and Regulated Lipid Metabolism Genes Expression

**Figure 2:**
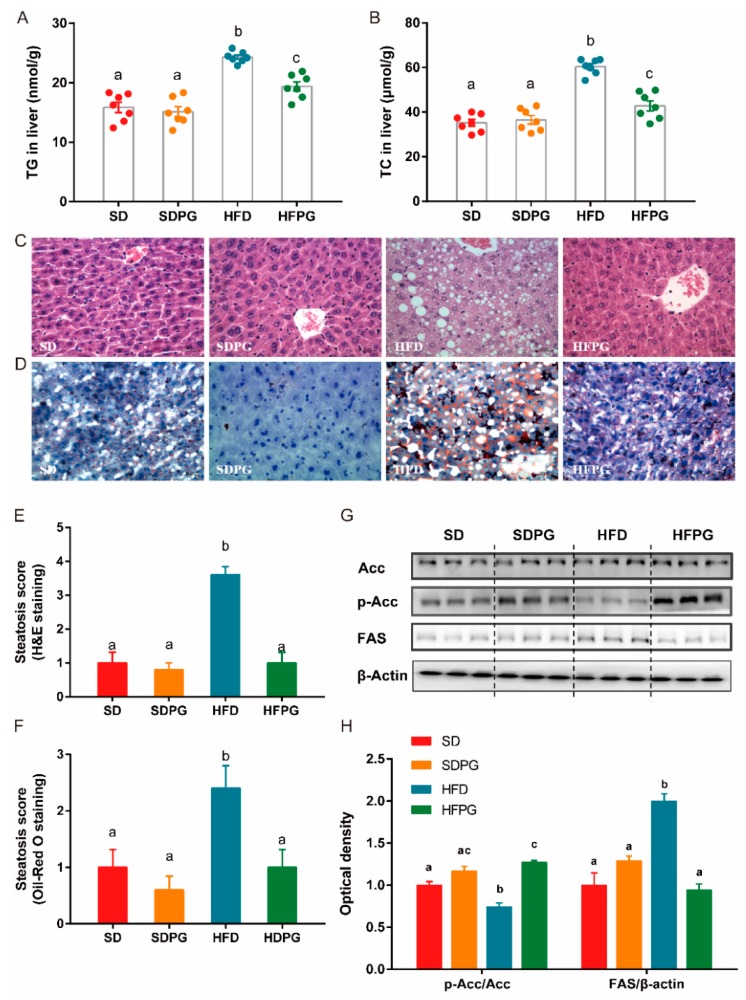
PG ameliorated steatosis and regulated lipid metabolism genes. (**A**) TG and (**B**) TC content in liver. (**C**,**D**) Hepatic steatosis determined by H&E (C) and Oil Red O (D) staining (scale: 50 μm) with steatosis scores shown in (**E**) and (**F**). (**G**) Western blot analysis for ACC, p-ACC, and FAS in liver tissues. (**H**) Fold change in expression of p-ACC/ACC and FAS/β-actin relative to SD group. TG, total triacylglycerol; TC, total cholesterol; ACC, acetyl-CoA carboxylase; p-ACC, phospho-ACC; FAS, fatty acid synthase. a, b, c means in the same bar without a common letter differ at *p* < 0.05.

We showed that changes in intrahepatic TG and TC in HFPG mice confirmed the beneficial effect of PG against HFD-induced excessive fat accumulation in the liver. Histological analysis further showed that livers of the HFD mice had prototypical lipid droplet accumulation (Figure 2C,D) and liver steatosis (Figure 2E,F), which were largely improved by PG to a similar level as in SD and SDPG mice.

To investigate the mechanism of PG protective effects against hepatic steatosis in HFD-fed mice, we examined the expression of several key genes involved in lipid metabolism. We showed that, with the supplementation of PG, phosphorylation of ACC was increased and expression of FAS was downregulated in HFPG mice, compared to HFD mice (Figure 2G,H). Meanwhile, PG had no significant effect on these genes in mice fed with the control diet (SDPG mice). All suggested that PG could effectively ameliorate hepatic steatosis and excessive fat accumulation induced by HFD.

### 3.3. PG Improved Insulin Sensitivity and Glucose Tolerance in HFD-Fed Mice

**Figure 3:**
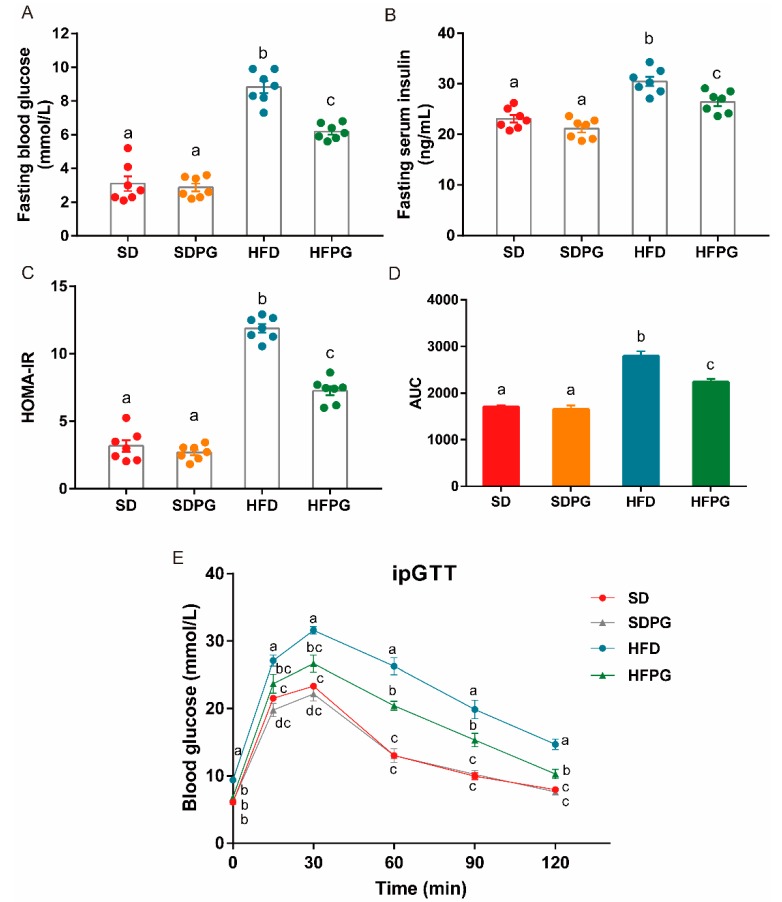
PG improved insulin sensitivity and glucose tolerance in HFD-fed mice. (**A**) Fasting blood glucose level. (**B**) Fasting serum insulin level. (**C**) HOMA-IR index. (**D**) Serum glucose level curves of ipGTT. (**E**) Area under the curve (AUC) of the blood glucose during ipGTT. a, b, c means in the same bar without a common letter differ at *p* < 0.05.

HFD mice exhibited elevated blood glucose and insulin levels resulting in increased values for HOMA-IR, which indicates a higher degree of insulin resistance, compared to SD mice. PG attenuated these effects in HFPG mice, resulting in similar levels of serum glucose and insulin compared to SD and SDPG mice (Figure 3A–C). Likewise, after 15 weeks of PG supplementation, glucose levels after the intraperitoneal injection and the AUC of blood glucose changing curve during the tested period in HFPG mice were significantly lower, compared to HFD mice (Figure 3D,E), suggesting that PG could improve insulin sensitivity and glucose tolerance.

### 3.4. PG Enhanced Hepatic Insulin Signaling Pathway PI3K/Akt/ GSK3ΒIn HFD Mice

**Figure 4:**
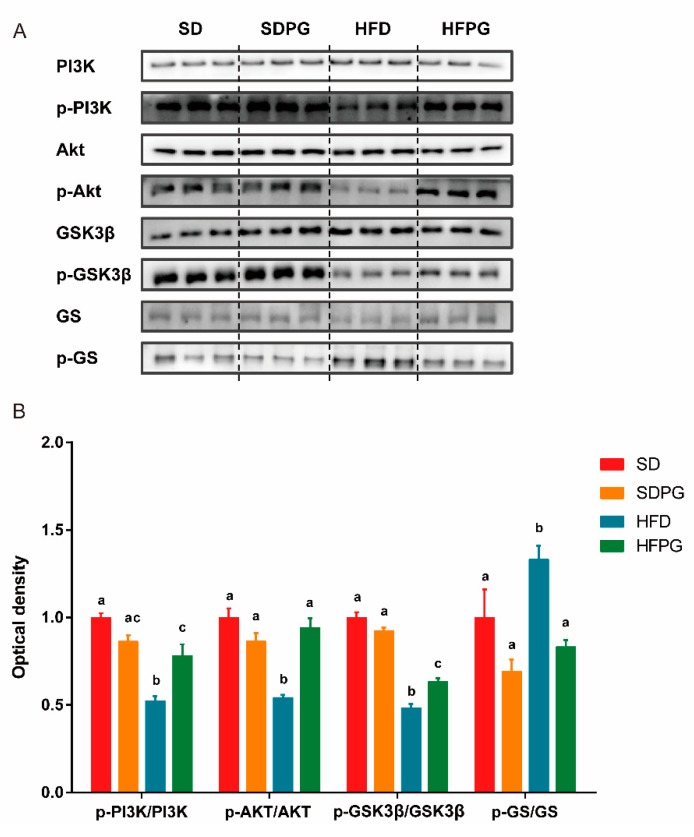
PG enhances hepatic insulin signaling PI3K/Akt/ GSK3β pathway in HFD-fed mice. (**A**) Western blot analysis for PI3K, p-PI3K, Akt, p-Akt, GSK3β, p-GSK3β, GS, and p-GS in liver tissues. (**B**) Fold change in expression of p-PI3K/PI3K, p-Akt/Akt, p-GSK3β/GSK3β, and p-GS/GS by densitometry relative to SD group. a, b, c means in the same bar without a common letter differ at *p* < 0.05.

The PI3K/Akt/GSK3β/GS pathway is a primary insulin signaling pathway affecting NAFLD development. We hence assessed the phosphorylation of PI3K and its downstream targets involved in insulin sensitivity in the liver among the four treatment groups (Figure 4A,B). HFD mice showed significantly impaired insulin signaling, including decrease of PI3K, Akt, GSK3β phosphorylation, as well as an increase in GS phosphorylation, compared with SD mice. The expression of hepatic total PI3K, Akt, GSK3β, and GS showed no significant difference between the four groups. PG supplementation significantly improved the phosphorylation of PI3K, Akt, and GSK3β and suppressed the phosphorylation of GS in mice fed HFD (HFPG) but not in mice fed with SD (SDPG). Taken together, we think PG may restore the impaired hepatic insulin signaling pathway caused by HFD.

### 3.5. PG Improved Hepatic Antioxidant Capacity and Reduced Inflammation in HFD-Fed Mice

The SOD, GSH-Px, and CAT enzymatic antioxidant activities are critical for defense against oxidative stress. Hence, we measured GSH content and antioxidative enzyme activities to evaluate the hepatic redox state (Figure 4A–D). The activities of CAT showed no significant difference among the 4 groups. Notably, PG efficiently reversed the activities of hepatic SOD, GSH-Px, and GSH content in HFD-fed mice to the same levels as in SD mice, except for GSH-Px. Interestingly, no significant difference in hepatic redox state was found between SDPG and SD mice.

HFD-induced oxidative stress can result in oxidative imbalance and lipid peroxidation. Hence, we next quantified the expression of CPT1 in mRNA level, as well as the essential antioxidative enzymes in protein level, including HO-1 and NQO1. We showed decreased expressions of CPT1, HO-1, and NQO1 by HFD were remarkably upregulated by PG to the similar levels in SD mice (Figure 4E,F).

**Figure 5:**
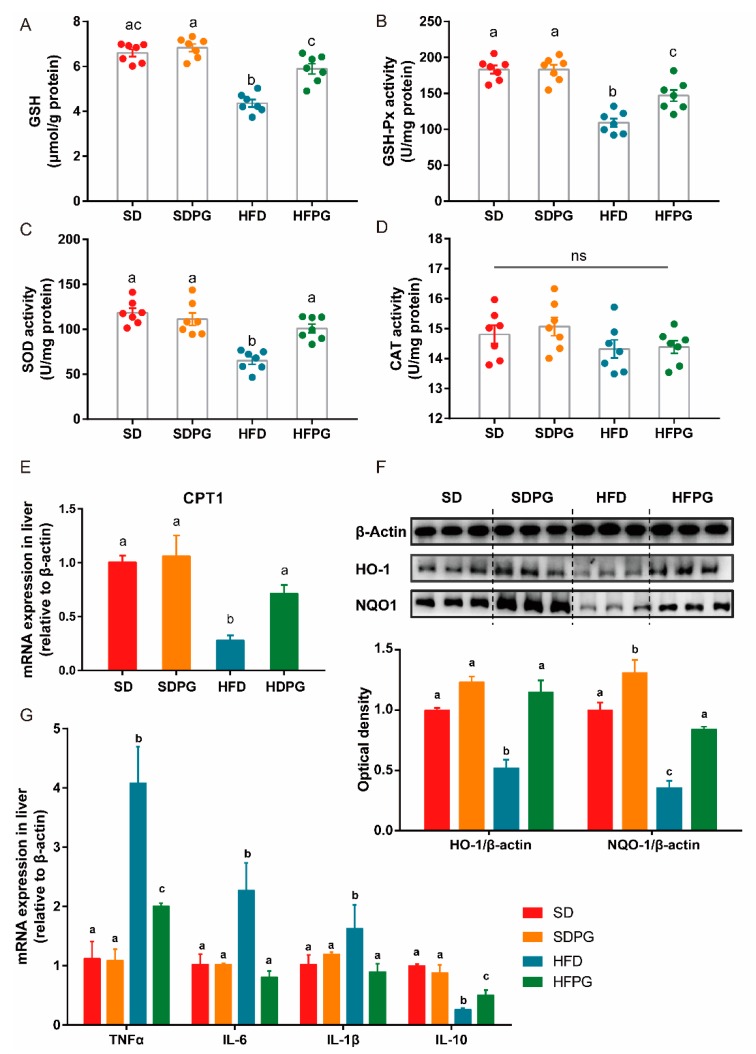
PG reduces hepatic oxidative stress in HFD-fed mice. (**A**) Liver GSH content. Activities of (**B**) GSH-Px, (**C**) SOD1, and (**D**) CAT, ns means no significant difference. (**E**) mRNA expression of CPT1 in liver analyzed by RT-qPCR. (**F**) Western blot analysis for HO-1, NQO1, and β-actin in liver tissues. (**G**) Fold change in relative expression of HO-1/β-actin and NQO1/β-actin in protein level. a, b, c means in the same bar without a common letter differ at *p* < 0.05.

Besides, we also found that HFD induced the inflammation in the liver. This effect was largely reduced by PG as based on the decreased expression of TNF-α, IL-6, and IL-1β, and the increase production of IL-10 in the liver (Figure 5G).

Taken together, PG could effectively ameliorate hepatic oxidative stress caused by HFD via increasing the expression of antioxidants and lipid oxidation enzymes and could improve hepatic inflammation by regulating inflammatory factors.

## 4. Discussion

NAFLD is a complex chronic metabolic disease and is a result of multiple interactions between genes [26]. Although the mechanism and causes of NAFLD are explained by some theories, including the two-hit theory, much remains unknown. In our study, we first confirmed the effect of PG on the “first hit” and investigated the crosstalk between genes involved in lipid and glucose metabolism in liver. Then, we evaluated the factors in “second hit”, including oxidation and inflammation levels. PG notably attenuated 16-week HFD-feeding induced hyperlipidemia, hepatic steatosis, insulin resistance, oxidative stress and inflammation in mice. Interestingly, SDPG mice, supplemented with PG, showed no significant difference to SD mice, suggesting that PG has no side effects, at least under normal energy intake. Currently, there is no proven, effective, and safe pharmacological therapy to treat NAFLD. Dietary intervention with whole PG, not an individual component or extraction, could also be an ideal way to help treat NAFLD, because of its convenience, relatively low costs, and minimal risk of side effects.

We showed that PG ameliorated hepatic steatosis, promoted ACC phosphorylate, and reduced FAS expression. ACC and FAS are key enzymes directly involved in fatty acid synthesis during lipogenesis and are known to be regulated by the AMPK signaling pathway. In agreement with our results, previous studies have shown that components or extractions of PG can mediate expression or activation of FAS, ACC, and other genes involved in the AMPK pathway to affect lipid metabolism in adipose and liver tissue in metabolic disease models [20,21,27]. Here, we demonstrated for the first time that dietary intervention by whole PG is efficient to improve hepatic steatosis via affecting expression of key enzymes.

In our study, PG significantly alleviated HFD induced insulin resistance by enhancing the PI3K/Akt/ GSK3β signaling pathway in the liver. The liver plays a pivotal role in maintaining blood glucose levels by balancing glucose storage and release via glycogen synthesis (glycogenesis) and breakdown (glycogenolysis) [28]. When blood glucose rises, insulin stimulates phosphorylation and activation of PI3K and Akt, regulating downstream GSK3β and GS, which are the main regulators of glycogen synthesis. Moreover, evidence indicated that the PI3K/Akt/GSK3β pathway is also closely related to lipogenesis [29,30] and circulating fatty acids [31]. Activation of the AMPK pathway can promote the phosphorylation of Akt and GSK3β [32]. We hypothesize that the crosstalk between hepatic lipid and glycogen metabolism may partially explain the universal finding that most NAFLD patients present insulin resistance [33,34].

Our results confirmed that PG could effectively reduce oxidative stress, which is also supported by previous studies using extractions or saponins from PG to ameliorate non-alcoholic steatohepatitis [22] or dimethyl nitrosamine-induced liver fibrosis [35]. Key enzymes, like GSH-Px and SOD, suppress or prevent the excessive oxidative stress in cells, acting as the first line of antioxidants defending from peroxidation of lipids induced by ROS [36]. CPT1 is the first and rate-limiting enzyme regulating fatty acid oxidation and can be inhibited by ACC via producing malonyl-CoA [37]. This can explain the decreased activity of ACC and increased expression of CPT1 in HFPG mice compared to HFD mice. In addition, recent research has identified Nrf2 and its downstream targets, HO-1 and NQO1, as key factors for combating hepatic oxidative stress by vegetables and the bioactive component [38,39], which were also shown to be upregulated by PG in this study. Increasing evidence indicates that PI3K/Akt are involved in the control of oxidative stress through the regulation of HO-1 and NQO1: previous research reported that stevia residue extract could increase antioxidative enzymes expression through the activation of the Akt/Nrf2/HO-1 pathway [40] in mice; bromocriptine was also reported to regulate Nrf2 activity and its downstream enzyme, NQO1, through the PI3K/Akt pathway [41] against oxidative stress. These findings confirmed oxidative stress as the second ‘hit’, following insulin resistance, which exacerbates NAFLD development. Overall, we suggest that PG can increase the activities of key antioxidants via increasing HO-1 and NQO1 expression, which may also be regulated by the activated PI3K/Akt pathway.

## 5. Conclusions

In summary, our results show that consumption of whole PG can attenuate hepatic steatosis, insulin resistance, oxidative stress, and inflammation, which are linked to regulated enzyme activities and gene expression in the PI3K/Akt/GSK3 or related pathways in HFD-induced NAFLD mice. Furthermore, we found no side effects of PG during the period of our experiment in four groups of mice, demonstrating the potential of whole PG, not only extractions or individual components, as a therapeutic strategy for NAFLD.

## Figures and Tables

**Table 1:** Serum TC, LDL-C, HDL-C levels and LDL-C/HDL-C ratio.

Serum Parameters	SD	SDPG	HFD	HFPG
TG (mg/dL)	0.27 ± 0.02 ^a^	0.26 ± 0.04 ^a^	0.42 ± 0.03 ^b^	0.28 ± 0.03 ^a^
TC (mg/dL)	3.02 ± 0.23 ^a^	2.93 ± 0.2 ^a^	5.2 ± 0.16 ^b^	3.89 ± 0.27 ^c^
HDL-C (mmol/L)	2.52 ± 0.19 ^a^	3.77 ± 0.12 ^a^	3.77 ± 0.12 ^b^	3.13 ± 0.18 ^c^
LDL-C (mmol/L)	0.42 ± 0.03 ^a^	0.38 ± 0.12 ^a^	1.18 ± 0.12 ^b^	0.7 ± 0.1 ^a^
LDL-C/HDL-C	0.18 ± 0.03 ^a^	0.17 ± 0.02 ^a^	0.31 ± 0.03 ^b^	0.22 ± 0.02 ^a^

TG, total triacylglycerol; TC, total cholesterol; LDL-C, low-density lipoprotein cholesterol; HDL-C, high-density lipoprotein cholesterol. Mean ± SEM, *n* = 8. One-way ANOVA, ^a, b, c^ means significant difference at *p* < 0.05.

## Supplementary Materials

The following are available online at https://www.mdpi.com/2072-6643/12/2/480/s1, Table S1: The composition of experimental diets. Table S2: The sequences of primers used in RT-qPCR assays.

## References

[1] Li J., Zou B., Yeo Y.H., Feng Y., Xie X., Lee D.H., Fujii H., Wu Y., Kam L.Y., Ji F. (2019). Prevalence, incidence, and outcome of non-alcoholic fatty liver disease in Asia, 1999–2019: A systematic review and meta-analysis. Lancet Gastroenterol. Hepatol..

[2] Younossi Z., Anstee Q.M., Marietti M., Hardy T., Henry L., Eslam M., George J., Bugianesi E. (2018). Global burden of NAFLD and NASH: Trends, predictions, risk factors and prevention. Nat. Rev. Gastroenterol. Hepatol..

[3] Day C.P., James O.F.W. (1998). Steatohepatitis: A tale of two “Hits”?. Gastroenterology.

[4] Sharma B.R., Kim H.J., Rhyu D.Y. (2015). Caulerpa lentillifera extract ameliorates insulin resistance and regulates glucose metabolism in C57BL/KsJ-db/db mice via PI3K/AKT signaling pathway in myocytes. J. Transl. Med..

[5] Kanai F., Ito K., Todaka M., Hayashi H., Kamohara S., Ishii K., Okada T., Hazeki O., Ui M., Ebina Y. (1993). Insulin-Stimulated GLUT4 Translocation Is Relevant to the Phosphorylation of IRS-1 and the Activity of PI3 Kinase. Biochem. Biophys. Res. Commun..

[6] Tuttle R.L., Gill N.S., Pugh W., Lee J.-P., Koeberlein B., Furth E.E., Polonsky K.S., Naji A., Birnbaum M.J. (2001). Regulation of pancreatic β-cell growth and survival by the serine/threonine protein kinase Akt1/PKBα. Nat. Med..

[7] Engin A. (2017). Non-alcoholic fatty liver disease. Advances in Experimental Medicine and Biology.

[8] Pirgon Ö., Bilgin H., Çekmez F., Kurku H., Dündar B.N. (2013). Association between insulin resistance and oxidative stress parameters in obese adolescents with non-alcoholic fatty liver disease. JCRPE J. Clin. Res. Pediatr. Endocrinol..

[9] Videla L.A., Rodrigo R., Araya J., Poniachik J. (2006). Insulin resistance and oxidative stress interdependency in non-alcoholic fatty liver disease. Trends Mol. Med..

[10] Rector R.S., Thyfault J.P., Uptergrove G.M., Morris E.M., Naples S.P., Borengasser S.J., Mikus C.R., Laye M.J., Laughlin M.H., Booth F.W. (2010). Mitochondrial dysfunction precedes insulin resistance and hepatic steatosis and contributes to the natural history of non-alcoholic fatty liver disease in an obese rodent model. J. Hepatol..

[11] Romero-Gómez M., Zelber-Sagi S., Trenell M. (2017). Treatment of NAFLD with diet, physical activity and exercise. J. Hepatol..

[12] Zheng X., Zhao M.-G., Jiang C.-H., Sheng X.-P., Yang H.-M., Liu Y., Yao X.-M., Zhang J., Yin Z.-Q. (2020). Triterpenic acids-enriched fraction from Cyclocarya paliurus attenuates insulin resistance and hepatic steatosis via PI3K/Akt/GSK3β pathway. Phytomedicine.

[13] Feng X., Yu W., Li X., Zhou F., Zhang W., Shen Q., Li J., Zhang C., Shen P. (2017). Apigenin, a modulator of PPARγ, attenuates HFD-induced NAFLD by regulating hepatocyte lipid metabolism and oxidative stress via Nrf2 activation. Biochem. Pharmacol..

[14] Park H.M., Park K.T., Park E.C., Kim S.I., Choi M.S., Liu K.H., Lee C.H. (2017). Mass spectrometry-based metabolomic and lipidomic analyses of the effects of dietary platycodon grandiflorum on liver and serum of obese mice under a high-fat diet. Nutrients.

[15] Kim H.L., Park J., Jung Y., Ahn K.S., Um J.Y. (2019). Platycodin D, a novel activator of AMP-activated protein kinase, attenuates obesity in db/db mice via regulation of adipogenesis and thermogenesis. Phytomedicine.

[16] Zhao X., Wang Y., Yan P., Cheng G., Wang C., Geng N., Wang X., Liu J. (2017). Effects of polysaccharides from platycodon grandiflorum on immunity-enhancing activity in vitro. Molecules.

[17] Kim J.Y., Hwang Y.P., Kim D.H., Han E.H., Chung Y.C., Roh S.H., Jeong H.G. (2006). Inhibitory effect of the saponins derived from roots of Platycodon grandiflorum on carrageenan-induced inflammation. Biosci. Biotechnol. Biochem..

[18] Lee K.J., Choi C.Y., Chung Y.C., Kim Y.S., Ryu S.Y., Roh S.H., Jeong H.G. (2004). Protective effect of saponins derived from roots of Platycodon grandiflorum on tert-butyl hydroperoxide-induced oxidative hepatotoxicity. Toxicol. Lett..

[19] Jeong C.H., Choi G.N., Kim J.H., Kwak J.H., Kim D.O., Kim Y.J., Heo H.J. (2010). Antioxidant activities from the aerial parts of Platycodon grandiflorum. Food Chem..

[20] Hwang Y.P., Choi J.H., Kim H.G., Lee H.-S., Chung Y.C., Jeong H.G. (2013). Saponins from Platycodon grandiflorum inhibit hepatic lipogenesis through induction of SIRT1 and activation of AMP-activated protein kinase in high-glucose-induced HepG2 cells. Food Chem..

[21] Khanal T., Choi J.H., Hwang Y.P., Chung Y.C., Jeong H.G. (2009). Protective effects of saponins from the root of Platycodon grandiflorum against fatty liver in chronic ethanol feeding via the activation of AMP-dependent protein kinase. Food Chem. Toxicol..

[22] Choi J.H., Jin S.W., Choi C.Y., Kim H.G., Kim S.J., Lee H.S., Chung Y.C., Kim E.J., Lee Y.C., Jeong H.G. (2017). Saponins from the roots of Platycodon grandiflorum ameliorate high fat diet-induced non-alcoholic steatohepatitis. Biomed. Pharmacother..

[23] Li D., Wang P., Wang P., Hu X., Chen F. (2018). Gut microbiota promotes production of aromatic metabolites through degradation of barley leaf fiber. J. Nutr. Biochem..

[24] Safari Z., Gérard P. (2019). The links between the gut microbiome and non-alcoholic fatty liver disease (NAFLD). Cell. Mol. Life Sci..

[25] Reagan-Shaw S., Nihal M., Ahmad N. (2008). Dose translation from animal to human studies revisited. FASEB J..

[26] Farrell G.C., Wong V.W.S., Chitturi S. (2013). NAFLD in Asia—As common and important as in the West. Nat. Rev. Gastroenterol. Hepatol..

[27] Lee C.E., Hur H.J., Hwang J.T., Sung M.J., Yang H.J., Kim H.J., Park J.H., Kwon D.Y., Kim M.S. (2012). Long-term consumption of Platycodi radix ameliorates obesity and insulin resistance via the activation of AMPK pathways. Evid. Based Complement. Altern. Med..

[28] Nordlie R.C., Foster J.D., Lange A.J. (1999). Regulation of glucose producing by the liver. Annu. Rev. Nutr..

[29] Krycer J.R., Sharpe L.J., Luu W., Brown A.J. (2010). The Akt-SREBP nexus: Cell signaling meets lipid metabolism. Trends Endocrinol. Metab..

[30] Porstmann T., Santos C.R., Griffiths B., Cully M., Wu M., Leevers S., Griffiths J.R., Chung Y.L., Schulze A. (2008). SREBP Activity Is Regulated by mTORC1 and Contributes to Akt-Dependent Cell Growth. Cell Metab..

[31] Gu Y., Gao L., Han Q., Li A., Yu H., Liu D., Pang Q. (2019). GSK-3β at the Crossroads in Regulating Protein Synthesis and Lipid Deposition in Zebrafish. Cell.

[32] Zheng T., Yang X., Wu D., Xing S., Bian F., Li W., Chi J., Bai X., Wu G., Chen X. (2015). Salidroside ameliorates insulin resistance through activation of a mitochondria-associated AMPK/PI3K/Akt/GSK3β pathway. Br. J. Pharmacol..

[33] Seppälä-Lindroos A., Vehkavaara S., Häkkinen A.-M., Goto T., Westerbacka J., Sovijärvi A., Halavaara J., Yki-Järvinen H. (2002). Fat Accumulation in the Liver Is Associated with Defects in Insulin Suppression of Glucose Production and Serum Free Fatty Acids Independent of Obesity in Normal Men. J. Clin. Endocrinol. Metab..

[34] Utzschneider K.M., Kahn S.E. (2006). Review: The role of insulin resistance in nonalcoholic fatty liver disease. J. Clin. Endocrinol. Metab..

[35] Choi J.H., Jin S.W., Kim H.G., Khanal T., Hwang Y.P., Lee K.J., Choi C.Y., Chung Y.C., Lee Y.C., Jeong H.G. (2013). Platycodi Radix attenuates dimethylnitrosamine-induced liver fibrosis in rats by inducing Nrf2-mediated antioxidant enzymes. Food Chem. Toxicol..

[36] Ighodaro O.M., Akinloye O.A. (2018). First line defence antioxidants-superoxide dismutase (SOD), catalase (CAT) and glutathione peroxidase (GPX): Their fundamental role in the entire antioxidant defence grid. Alex. J. Med..

[37] Abu-Elheiga L., Matzuk M.M., Abo-Hashema K.A.H., Wakil S.J. (2001). Continuous fatty acid oxidation and reduced fat storage in mice lacking acetyl-coa carboxylase 2. Science.

[38] Zhang X., Ji R., Sun H., Peng J., Ma X., Wang C.Y., Fu Y., Bao L., Jin Y. (2018). Scutellarin ameliorates nonalcoholic fatty liver disease through the PPARγ/PGC-1α-Nrf2 pathway. Free Radic. Res..

[39] Xu Z., Wang S., Ji H., Zhang Z., Chen J., Tan Y., Wintergerst K., Zheng Y., Sun J., Cai L. (2016). Broccoli sprout extract prevents diabetic cardiomyopathy via Nrf2 activation in db/db T2DM mice. Sci. Rep..

[40] Zhao L., Yang H., Xu M., Wang X., Wang C., Lian Y., Mehmood A., Dai H. (2019). Stevia residue extract ameliorates oxidative stress in D-galactose-induced aging mice via Akt/Nrf2/HO-1 pathway. J. Funct. Foods.

[41] Lim J.H., Kim K.M., Kim S.W., Hwang O., Choi H.J. (2008). Bromocriptine activates NQO1 via Nrf2-PI3K/Akt signaling: Novel cytoprotective mechanism against oxidative damage. Pharmacol. Res..

