# Supplementary material for: Dietary Platycodon grandiflorus Attenuates Hepatic Insulin Resistance and Oxidative Stress in High-Fat-Diet Induced Non-Alcoholic Fatty Liver Disease
Source: Nutrients. 2020 Feb 14;12(2):480. doi: 10.3390/nu12020480 (PMC7071327; doi:10.3390/nu12020480)
Supplement: Supplementary file 1 [file nutrients-12-00480-s001.pdf]

Supplementary Table S1. The composition of experimental diets.

Ingredient	Standard chow Diet (SD)		High-fat Diet (HFD)	
	g%	kcal%	g%	kcal%
Casein, 30 Mesh	18.96	19.72	25.85	19.72
L-Cystine	0.28	0.30	0.39	0.30
Corn Starch	29.86	31.06	0.00	0.00
Maltodextrin 10	3.32	3.45	16.15	12.32
Sucrose	33.18	34.51	8.89	6.78
Cellulose, BW200	4.74	0	6.46	0.00
Soybean Oil	2.37	5.55	3.23	5.55
Lard	1.90	4.44	31.66	54.35
Mineral Mix S10026	0.95	0	1.29	0.00
Dicalcium Phosphate	1.23	0	1.68	0.00
Calcium Carbonate	0.52	0	0.71	0.00
Potassium Citrate, 1 H2O	1.56	0	2.13	0.00
Vitamin Mix V10001	0.95	1	1.29	0.99
Choline Bitartrate	0.19	0	0.26	0.00
**Energy (kcal/g diet)**	3.85		5.24	

Supplementary Table S2. The sequences of primers used in RT-qPCR assays.

Genes	Forward Primer	Reverse Primer
IL-6	5’-CCGGAGAGGAGACTTCAC-3’	5’-TCCACGATTTCCCAGAG-3’
IL10	5’-GCTCTTACTGACTGGCATGAG-3’	5’-CGCAGCTCTAGGAGCATGTG-3’
IL-1β	5’-TTGAAGAAGAGCCCATCCTC-3’	5’-CAGCTCATATGGGTCCGAC-3’
TNFα	5’-TAGCCAGGAGGGAGAACAGA-3’	5’-TTTTCTGGAGGGAGATGTGG-3’
CPT1	5’-GACTCCGCTCGCTCATTCC-3’	5’-GACTGTGAACTGGAAGGCCA-3’
